# Medial Compartment Knee Osteoarthritis Altered Tibiofemoral Joint Kinematics and Contact Pattern During Daily Weight‐Bearing Extension

**DOI:** 10.1111/os.70023

**Published:** 2025-03-18

**Authors:** Zheng Jiang, Axiang He, Nan Zheng, Yanjie Mao, Weiming Lin, Xiaoyin Zhang, Han Guo, Yuyan Liu, Tsung‐Yuan Tsai, Wanjun Liu

**Affiliations:** ^1^ Department of Orthopedics Shanghai Sixth People's Hospital Affiliated to Shanghai Jiao Tong University School of Medicine Shanghai China; ^2^ School of Biomedical Engineering & Med‐X Research Institute Shanghai Jiao Tong University Shanghai China; ^3^ Shanghai Ocean University Shanghai China

**Keywords:** contact pattern, kinematics, medial compartment knee osteoarthritis, tibiofemoral joint

## Abstract

**Objective:**

With the advancement of digital orthopedics, the growing prevalence of medial compartment knee osteoarthritis (MCKOA) and the widespread adoption of knee‐preserving surgical techniques have heightened new interest in predicting the onset of MCKOA and promoting surgical outcomes. This study was to clarify the differences in kinematics and contact patterns between the MCKOA knee and its native sides during knee extension.

**Methods:**

From March 2023 to June 2024, thirty‐two patients who suffered from unilateral MCKOA, with their contralateral extremities asymptomatic and intact, were enrolled in this descriptive research. Three‐dimensional models were created from computed tomography scans, and all patients performed continuous stair climbing under the surveillance of a dual fluoroscopic imaging system (DFIS) to determine the accurate 6‐degrees‐of‐freedom (6‐DOF) of their medial OA knees and the contralateral knees. The volume penetration centers between tibial and femoral cartilage models were defined as contact centers. All measured parameters were tested for significant differences using the Wilcoxon Rank‐Sum test.

**Results:**

Compared to native knees, the MCKOA tibia showed increased flexion (mean 3.6°) and varus rotation (mean 1.6°), with more posterior (mean 1.4 mm), lateral (mean 1.2 mm) and proximal translations (mean 0.5 mm) relative to the femur during extension (*p* < 0.05). The tibiofemoral contact patterns on the medial and lateral tibial plateau of the MCKOA knee both shifted more medially (mean 1.4 mm and 1.3 mm, respectively, *p* < 0.05) than the native side, which was consistent with the lateral translations observed in 6‐DOF.

**Conclusion:**

Our findings offer valuable insights into the in vivo kinematics of MCKOA knee, its tibiofemoral joint (TFJ) and contact pattern. In MCKOA knees, the tibia exhibited increased flexion and varus rotation, along with more posterior, lateral, and proximal translation relative to the femur compared to the native side during extension. These changes aligned with the more medial shifts in contact patterns of the tibial plateau on the MCKOA side. These findings provide data support for the digital diagnosis and treatment of MCKOA.

## Graphical abstract

Under the surveillance of dual fluoroscopic imaging system (DFIS), the MCKOA side tibia showed more flexed and varus rotations and more posterior, lateral, proximal translations than its native side during daily extension, consistent with the extra medial shifts of contact patterns on MCKOA side tibial plateau.

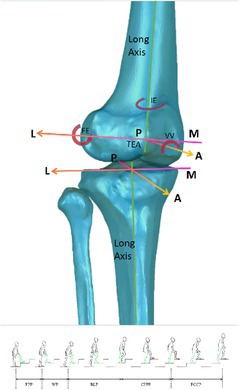

## 1 Introduction

The incidence of knee osteoarthritis (KOA) is increasing year by year due to the aggravation of an aging population and an increase in the global obesity rate [1]. Among different KOA types, medial compartment KOA (MCKOA) refers to the medial compartment of the knee joint as the main affected site, which manifests as narrowing of the medial joint space, erosion of medial articular cartilage, varus deformity of the knee joint, and ‘O’ type leg changes [2]. Isolated MCKOA tends to occur as the early stage of KOA. Nowadays, MCKOA is the most common form of KOA demanding knee preservation surgeries [3]. Currently, the objective evidence for diagnosing MCKOA in clinical practice relies on static parameters such as X‐rays and CT scans. However, these static parameters are influenced by factors like patient postures, leading to failure in the early detection and diagnosis of MCKOA in clinical practice. Besides, the outdated equipment and small sample sizes of previous kinematic studies made their conclusions inadequate to meet the needs of modern digital orthopedics, such as computer‐assisted navigation or robotic surgeries. Clarifying the in vivo kinematic characteristics of MCKOA knees is essential for the detection, diagnosis, and digital surgeries of MCKOA.

In vivo kinematics plays an essential role in observing normal or osteoarthritic (OA) knees in motion. The knee moves through three dimensions, with 6 degrees of freedom [4] (6‐DOF) and the knee motion is proved to be related to the knee joint cartilage thickness. Because excessive motion or an offset in rotation may lead to cartilage thinning [5]. It was reported that different kinematic modifications affected knee cartilage mechanics in individuals with medial tibiofemoral KOA [6]. Previous research on normal knees has indicated that during stair ascent, the tibiofemoral joint (TFJ) underwent a distinct pattern of flexion. At the heel‐strike phase, the TFJ was highly flexed and internally rotated, transitioning to extension and external rotation for the majority of the stance phase [7]. Nagano Y et al. [8] compared the knee kinematic variables between OA patients and normal subjects and found that early KOA stage patients showed decreased tibial rotation excursion, while their flexion‐extension course was almost identical to that of normal subjects. To our best knowledge, no kinematic studies have yet compared the MCKOA knee with its contralateral, unaffected side under the same physiological conditions. For optimal restoration of postoperative function, it is crucial to elucidate the distinct movement characteristics of the MCKOA knee when compared to its healthy counterpart.

The contact patterns can directly indicate the zones of contact compressive force positioned in knee compartments, where the wear of cartilage tends to occur. It was confirmed that the initial cartilage damage was associated with abnormal kinematics that shifted load bearing. Then, with cartilage breakdown, the disease progressed more rapidly as load increased [9]. Additionally, contact patterns could serve as a critical indicator for post‐operative loading consideration [10]. To date, the contact patterns of MCKOA were reported contradictorily. Katsutoshi Nishino et al. [11] found that in severe MCKOA knees, the loading axis of the knee, which was located on the proximal tibial surface, was positioned not only more medially but also posteriorly compared to that in normal knees during static standing. This result was consistent with tibial cartilage wear and bony defect locations observed in medial KOA [12]. However, Satoshi Hamai et al. [13] used a high‐resolution flat panel x‐ray detector to observe the motion of knees with MCKOA, finding the contact positions showed mild anterior femoral translation with knee extension from 80° to 20°. Ashok Rajgopal et al. [14] conducted an observation on the anatomic distribution of varus OA knees cartilage pathology and found that in an overwhelming 99.5% of cases, tibial surface wear was localized to the antero‐medial third of the tibial plateau. Therefore, there is still a lack of a precise quantitative study to clarify the contact patterns differences between MCKOA knees and native knees.

The DFIS technique is widely recognized as the most accurate non‐invasive method for tracking in vivo dynamic motion, offering a high level of precision with an accuracy of approximately 0.1 mm and 0.3° [15, 16]. This technique has been extensively utilized for observing and analyzing in vivo knee joint kinematics [17, 18]. However, the sample sizes in previous studies have often been too small (less than 10 [19]) to produce reliable and consistent findings. Among various functional activities, climbing stairs is considered one of the most physically demanding tasks under weight‐bearing conditions [20]. Both 6‐DOF and contact patterns are commonly employed in digital surgeries. Thus, accurately identifying the kinematics of the femur and tibia by DFIS during staircase weight‐bearing extension is of significant clinical relevance, particularly for the diagnosis and treatment of medial compartment knee osteoarthritis (MCKOA).

The purposes of this study were to clarify the differences of (i) knee 6‐DOF kinematics and (ii) the tibiofemoral contact patterns between MCKOA knees and their native sides, in order to provide the data support for the digital detection, diagnosis, and treatments of MCKOA.

## 2 Materials and Methods

### 2.1 Research Subject Selection

From March 2023 to June 2024, 32 patients (7 male and 25 female) suffered from unilateral MCKOA (14 left and 18 right) with their contralateral extremities asymptomatic and intact who were recruited in this descriptive research, complying with the regulations of the Institutional Review Board (IRB No. *2023‐KY‐071(K)*). A post‐hoc statistical benefit‐volume analysis was performed using G*Power 3.1 (Universität Kiel, Kiel, Germany), and the 32 MCKOA patients included in this study had effect sizes above 0.95. The average age was 60.7 ± 3.7 years old and the average body mass index was 28.5 ± 3.6 kg/m^2^. All patients prepared to receive unilateral surgical treatments due to one of their varus knees with early KOA (Kellgren‐Lawrence [21] (K‐L) grade I–III). The inclusion criteria were the clinical diagnostic standards for unilateral MCKOA, with the contralateral knee being healthy. The exclusion criteria were a previous knee trauma and surgery history in either lower limb or any other diseases affecting cooperation, like neuropsychiatric diseases.

### 2.2 Kinematic Data Analysis

**FIGURE 1:**
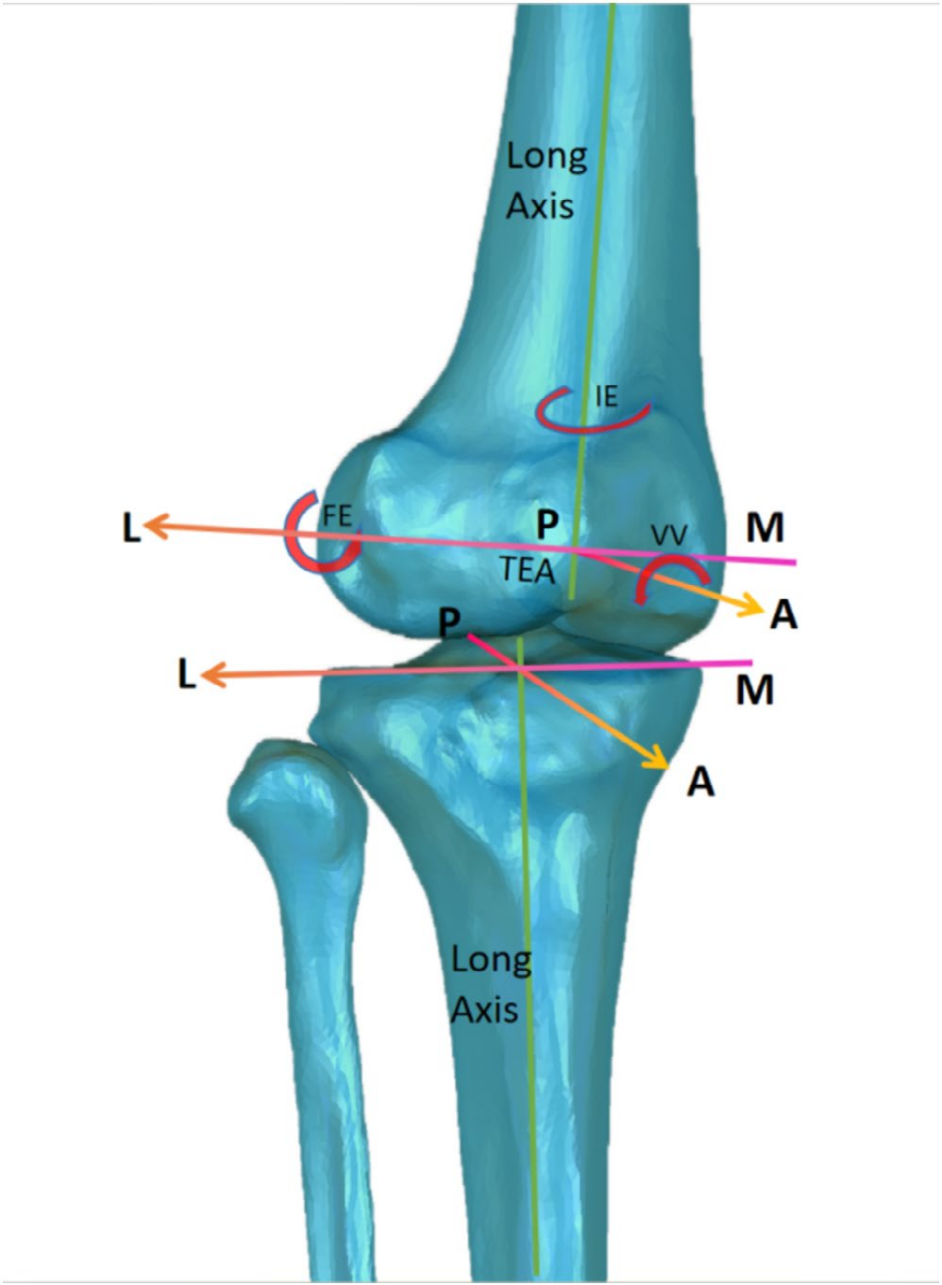
Coordinate systems used to quantify relative motions of the MCKOA tibia and femur in the current study: The tibial 6 degrees‐of‐freedom (6‐DOF) relative to the femur was quantified during stair climbing motion, which was described as flexion‐extension (FE), internal‐external adduction (varus‐vaglus, VV), internal‐external rotation (IE), anterior–posterior translations (AP), medial‐lateral translations (ML), and superior–inferior translations (SI). The motions like anterior translation, lateral translation, and superior translation are considered positive, and the motions rotating clockwise around three axes like extension, internal adduction (varus), and internal rotation are also considered positive.

Each patient underwent CT scans (Sensation 64, Siemens, Germany) for both lower limbs. All patients received MRI scanning for both knees using a T1‐weighted fat‐suppression sequence (3.0 T, Philips, Holland; sequence settings refer to previous studies [22]). The T1 sequence was used to reconstruct a three‐dimensional (3D) cartilage model for each participant's knee to match with CT models of the patella and femur. The CT and T1 MRI images were input into Amira (Thermo Fisher Scientific, Rockford IL, USA) to build 3D surface models of the femur and tibia with cartilages. The bone coordinate systems were built referring to the definition recommended by the International Society of Biomechanics and previous studies of our team [16, 23, 24] (Figure 1).

**FIGURE 2:**
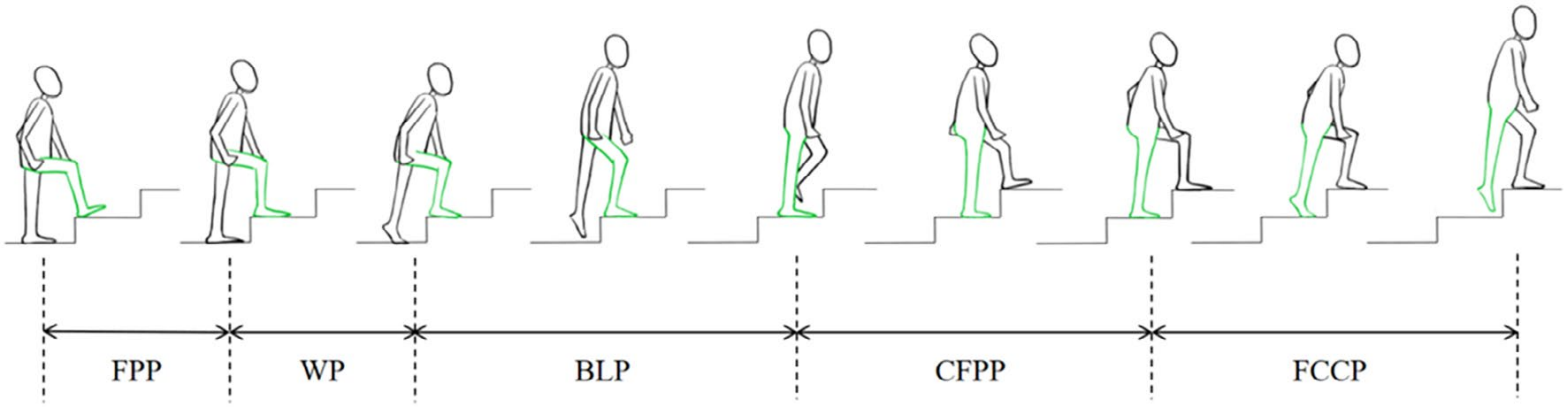
A complete gait cycle scheme during stair climbing including a support phase: Weight‐bearing Phase (WP), Body Lifting Phase (BLP), Continuous Forward Propulsion Phase (CFPP) and a swing phase: Foot Contour Clearance Phase (FCCP) and Foot Placement Phase (FPP) [26].

DFIS technique was used to quantify the tibial motion relative to the femur during stair climbing and static standing for bilateral knees of each patient. The stair riser height was set at 14 cm, which is one of the outdoor safety standards used in China and North America [25]. The patients were asked to climb three stairs continuously under the surveillance of DFIS (TAO image, China) in the national center for orthopedics (China). The knee joint was imaged using 30 pulsed snapshots per second (8 ms/pulse) by DFIS as the patients climbed the second stair. When taking DFIS dynamic images, a high‐frame‐rate force measurement table was arranged under the second step. The synchronized force signals were processed using a 5 Hz Butterworth low‐pass filter (1000 Hz, Bertec, USA), and the heel‐striking to toe‐off moments of the subject's foot were extracted as the cycle of motion up the stairs (Figure 2). The body‐lifting phase was picked out and measured in this study as a motion cycle of weight‐bearing extension. The 3D surface model and 2D DFIS images were imported into the customized software (MATLAB, 2023b, USA), adjusting the models to match the bones' outlines on each perspective image to reconstruct the spatial position of bones during stair climbing.

The femoral 6‐DOF relative to the tibia were quantified during extension motion, which were described as flexion‐extension (FE) coupled with the internal‐external (IE), varus‐valgus (VV) rotation, and the antero‐posterior (AP), medio‐lateral (ML), and proximo‐distal (PD) translations. The TFJ translations were displayed in terms of the femur relative to the tibia as the weight‐bearing extension was a closed‐chain motion, while the TFJ rotations were displayed in terms of the tibia relative to the femur. The motion cycle was divided into 100 equal segments, and data was collected at each 10% interval for analysis. Besides, the determination of tibiofemoral articular cartilage contact points was by the centroid of the overlapping areas of the tibiofemoral cartilage surfaces, which were normalized relative to the tibial plateau and patellar widths.

The native knee was considered to be the reference for the MCKOA side, as the difference in knee kinematics among individuals is significantly larger than that between bilateral knees for human beings [27]. Furthermore, there is a growing consensus that functional recovery counts more than mechanical axis alignment (MA) in knee corrective procedures [28]. The native sides of these patients with unilateral MCKOA were less deformed, less painful, and more well‐functioning than their MCKOA sides, which makes them the most suitable references for evaluation.

All measured parameters were tested for significant differences using the Wilcoxon Rank‐Sum test by MATLAB. A *p* value less than 0.05 is considered to indicate a significant difference.

## 3 Results

### 3.1 Differences in 6‐DOF Kinematics

**FIGURE 3:**
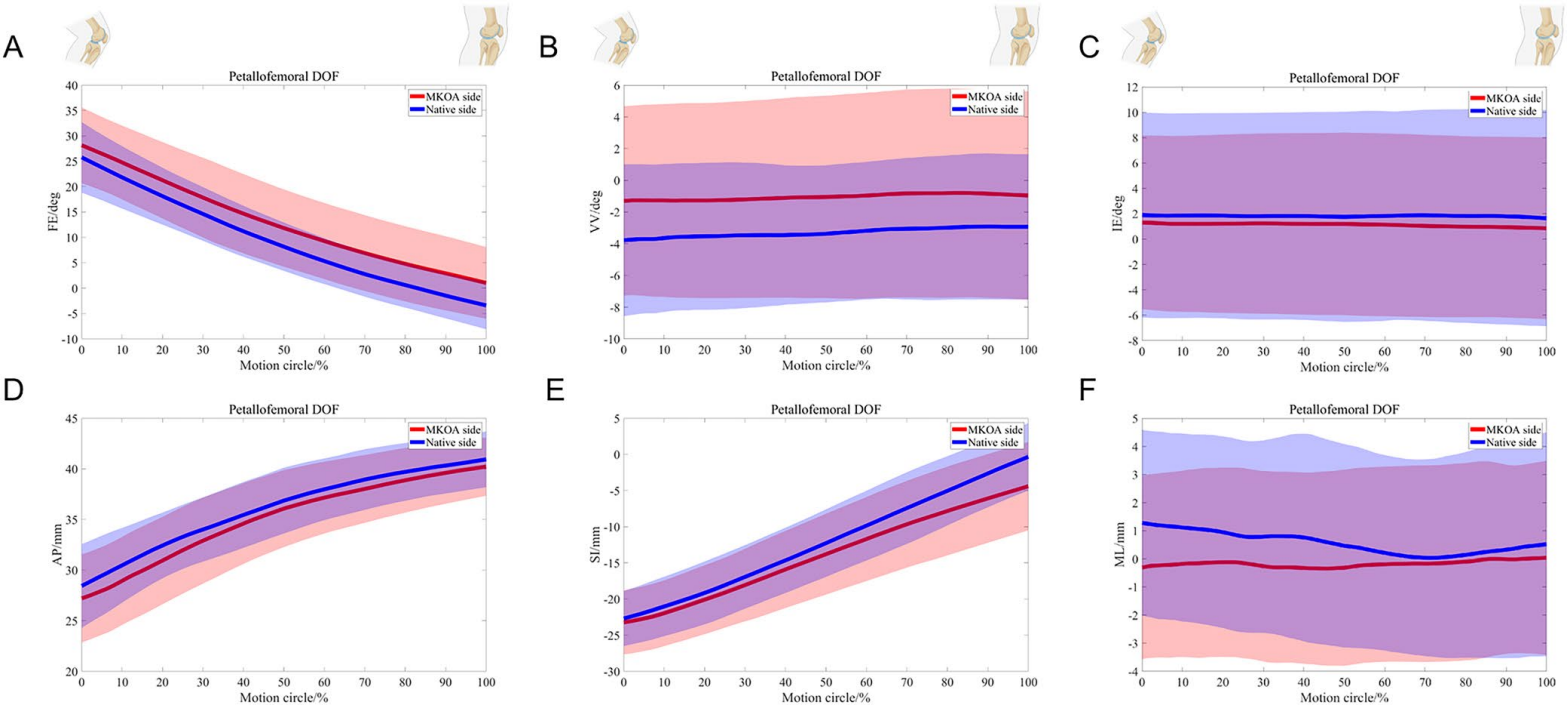
Comparison of 6‐DOF kinematics between two‐sided knees during daily weight‐bearing extension with confidence interval regions: (A) The flexion‐extension (FE) rotation trends, (B) The varus‐valgus (VV) rotation trends, (C) The internal‐external (IE) rotation trends, (D) The anterior–posterior (AP) translation trends, (E) The proximal–distal (PD) translation trends, and (F) The medial–lateral (ML) translation trends.

For the 6‐DOF of the tibia in relation to the femur, the tibia on the side with MCKOA clinical evidence was more flexed (mean 3.6°) and posterior (mean 1.4 mm) during most extension than the native side. The extra flexion of the MCKOA side increased as the knee extended. During 20%–90% of the motion cycle, the tibia on the MCKOA side was more varus (mean 1.6°) and translated more laterally (mean 1.2 mm) near the end of the motion cycle than that on the native side. At the first 40% of the motion cycle, the MCKOA tibia continued to show more proximal shifts (mean 0.5 mm) than the native side. (Table 1 and Figure 3).

**TABLE 1:** The 6‐DOF differences of TFJ between native and MCKOA knees during extension (Mean ± SD).

6‐DOF	0%	10%	20%	30%	40%	50%	60%	70%	80%	90%	100%
FE	1.29 ± 5.81	1.5 ± 6.36	1.65 ± 7.08	1.42 ± 7.69	1.4 ± 7.29*	1.6 ± 6.6*	2.2 ± 6.15*	2.74 ± 6.26*	3.31 ± 6.53*	4.33 ± 6.99*	5.31 ± 7.45*
VV	1.15 ± 3.51	1.13 ± 3.43	1.23 ± 3.24*	1.41 ± 3.11*	1.73 ± 3.01*	1.66 ± 3.16*	1.69 ± 3.18*	1.75 ± 3.16*	1.8 ± 3.26*	1.79 ± 3.26*	1.61 ± 3.22*
IE	2.15 ± 6.72	2.07 ± 6.63	2.16 ± 6.74	2.12 ± 6.62	2.2 ± 6.59	2.15 ± 6.7	2.16 ± 7.07	2.06 ± 7.27	2.07 ± 7.4	2.15 ± 7.66	1.91 ± 7.84
AP	0.98 ± 3.06	1.29 ± 3.1*	1.39 ± 3.18*	1.4 ± 3.31*	1.48 ± 3.24*	1.5 ± 2.91*	1.39 ± 3.19*	1.53 ± 3.32*	1.52 ± 3.35*	1.26 ± 3.23*	1.14 ± 3.49
PD	−0.56 ± 1.46*	−0.51 ± 1.31*	−0.47 ± 1.23*	−0.41 ± 1.28*	−0.34 ± 1.37*	−0.39 ± 1.46	−0.36 ± 1.46	−0.36 ± 1.4	−0.39 ± 1.41	−0.45 ± 1.43*	−0.37 ± 1.43
ML	−0.67 ± 3.19	−0.57 ± 2.87	−0.91 ± 2.61	−1.04 ± 2.44*	−1.19 ± 2.38*	−1.21 ± 2.28*	−1.29 ± 2.23*	−1.34 ± 2.25*	−1.27 ± 2.39*	−1.2 ± 2.34*	−1.01 ± 2.31*

* *p* value < 0.05.

### 3.2 Differences in TFJ Contact Patterns

**FIGURE 4:**
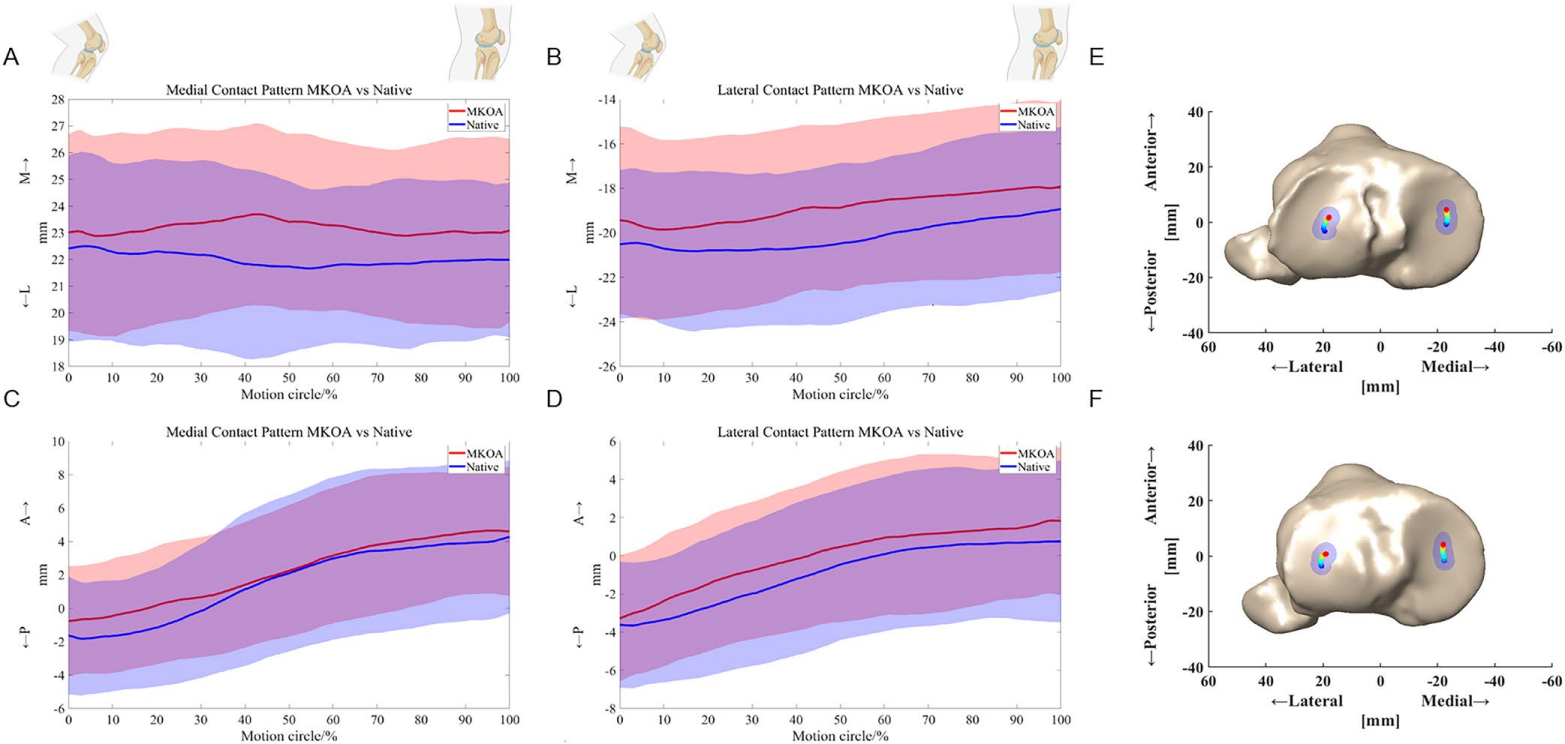
Comparison of contact patterns between two sides during daily weight‐bearing extension with confidence interval regions: TFJ contact center locations on the medial and lateral tibial plateau on ML translation trends (A, B) and AP translation trends (C, D). Locations of the TFJ contact pattern displayed on the tibial plateau of the MCKOA side (E) and native side (F) as viewed from the superior direction by a plane coordinate system.

During most motion cycles of weight‐bearing extension, contact locations on both sides showed a medial pivot pattern. The contact centers on the medial and lateral tibial plateau both shifted more medially (mean 1.4 mm and 1.3 mm, respectively) on the MCKOA side than on the native side, meaning that the tibia on the MCKOA side shifted more laterally relative to the femur. The extra anterior translation of contact patterns on the MCKOA side was also detected, but the differences were not significant (Table 2 Figure 4).

**TABLE 2:** The contact pattern differences on the medial tibial plateau (MTP) and lateral tibial plateau (LTP) between native and MCKOA knees during extension (Mean ± SD).

Tibial plateau	0%	10%	20%	30%	40%	50%	60%	70%	80%	90%	100%
MTP‐AP	0.87 ± 3.73	1.19 ± 3.48	1.33 ± 3.61	0.83 ± 3.88	0.27 ± 4.02	0.15 ± 4.02	0.17 ± 4.12	0.36 ± 4.17	0.47 ± 4.17	0.64 ± 4.12	0.32 ± 4.15
MTP‐ML	0.6 ± 3.59	0.63 ± 3.56	0.89 ± 3.54	1.19 ± 3.51	1.8 ± 3.46*	1.67 ± 3.25*	1.5 ± 3.04*	1.2 ± 3.1*	1.05 ± 3.22	1.06 ± 3.29*	1.1 ± 3.16*
LTP‐AP	0.33 ± 3.31	1 ± 3.35	1.21 ± 3.56	1.21 ± 3.69	1.04 ± 3.88	0.93 ± 3.95	0.86 ± 4.05	0.7 ± 4.15	0.69 ± 3.96	0.76 ± 3.86	1.08 ± 4.05
LTP‐ML	1.08 ± 3.77*	0.86 ± 3.72*	1.15 ± 3.75*	1.34 ± 3.65*	1.72 ± 3.61*	1.61 ± 3.68*	1.57 ± 3.59*	1.38 ± 3.64*	1.23 ± 3.82*	1.22 ± 3.83*	1.02 ± 3.77*

* *p* value < 0.05.

## 4 Discussion

This study investigated the characteristic motion patterns of the MCKOA knee during weight‐bearing extension by using 6‐DOF and contact patterns. First, The tibia on the MCKOA knee showed more flexed, varus rotation and more posterior, lateral, proximal translation related to the femur than native knee motion. Then, the contact centers of the medial and lateral tibial plateau on the MCKOA side both shifted more medially than the native side.

### 4.1 Differences in 6‐DOF Kinematics

The 6‐DOF kinematics of varus knees with KOA symptoms in our study were mostly consistent with previous findings. Rob et al. [29] used inertial sensor technology to discriminate movement behavior between healthy controls and patients with KOA and reported that OA knees had significantly less knee flexion during the forward lunge, single‐leg squat, and ascent and descent of stairs. Furthermore, Kenji Hoshi et al. [19] found that knee varus rotation and tibial lateral translation increased from the unloading phase to the weight‐loading phase, resulting in no change in internal rotation of the tibia. Our findings aligned with those of previous studies, which indicated that the tibia on the MCKOA side exhibited greater flexion [30] along with increased varus angles and lateral shifts during knee extension. Such lateral shifts were proved to be related to the progress of MCKOA [31]. These factors have been shown to elevate medial knee loads, thereby contributing to the development of MCKOA [32]. However, our study discovered extra proximal (mean 0.5 mm) and posterior (mean 1.4 mm) translations of the MCKOA tibia. Two types of accounts could be proposed for such new findings. The first account assumed that the TFJ cartilage thinning of the MCKOA knee led to the more distal position of the femur, whereas the second account assumed that the advanced DFIS equipment and large sample size of our observation might allow recognition of significant differences more easily.

### 4.2 TFJ Contact Patterns

The description of knee joint extension using the contact patterns may have interesting clinical implications. Scarvell JM et al. [32] used MRI scans and found that contact in the lateral and medial compartments of OA knees was more anterior on the tibial plateau than in healthy knees during knee extension, and this anterior contact pattern was associated with the severity of OA. Our observation also detected the extra anterior translation of contact patterns on the MCKOA side, but the differences were not significant. A possible explanation for this might be that the morphological changes of MCKOA knees were not that serious. Besides, the observation of Satoshi Hamai et al. [13] showed that from 100° to 120° flexion, KOA contact locations showed a medial pivot pattern similar to normal knees. In this study, extra medial shifts of contact centers on MCKOA knees were detected compared to the native knees, which accorded with the extra tibial lateral translation observed in the 6‐DOF of the MCKOA knee. The abnormal distribution of contact points in MCKOA may lead to exacerbated cartilage wear at corresponding locations on the tibial plateau and injury to adjacent soft tissues.

### 4.3 Strengths and Limitations

The limitations of this study should be mentioned. The study only focused on the extension phase in daily stair‐climbing movement, which is a type of middle‐amplitude flexion movement. Other knee joint movements, such as running, walking, squatting, etc., should be measured in further studies. In addition, the native side limbs of these patients may not be in perfect healthy condition, either because there are no clinical symptoms or because the varus degrees are mild that leave them free from surgery. So, adding a group of normal knees might be helpful to make our observation more comprehensive. Despite these limitations, this study utilized advanced DFIS demonstrating the in vivo kinematics and contact patterns of the MCKOA knees, which provide a reference for the improvement of related surgeries.

### 4.4 Prospects of Clinical Application

The findings of this study provide a reference for the early diagnosis and digital surgical treatment of MCKOA (or early KOA). Additionally, the dynamic motion characteristics of the MCKOA knee joint may offer directions for the prediction and prevention of the knee joints at potential risk of degeneration. Furthermore, to address the limitations of static imaging parameters, this study enriches the data foundation for dynamic evaluation in orthopedic diseases based on DFIS.

## 5 Conclusion

Our findings provide valuable insights into the in vivo kinematics of MCKOA knees, together with their TFJ contact patterns. The tibia in MCKOA knees showed increased flexion, varus rotation, and greater posterior, lateral, and proximal translation relative to the femur compared to the native side during extension, consistent with the medial shifts of contact centers on the MCKOA tibial plateau. These findings provide the reference for the digital detection, diagnosis, and surgical treatments of MCKOA and also offer a practical and objective method for personalized knee evaluation and KOA prevention.

## Author Contributions

**Zheng Jiang:** conceptualization, methodology, data collection and curation, formal analysis, investigation, writing original draft, review and editing. **Axiang He:** clinical operations, validation, supervision, review and editing, funding acquisition. **Nan Zheng:** conceptualization, methodology, formal analysis, investigation, visualization, review and editing. **Yanjie Mao:** conceptualization, clinical operations, data curation, investigation. **Weiming Lin:** investigation, model reconstruction. **Xiaoyin Zhang:** formal analysis, investigation, methodology. **Han Guo:** formal analysis, investigation, methodology. **Yuyan Liu:** formal analysis, investigation. **Tsung‐Yuan Tsai:** conceptualization, guidance in DFIS processing, language polishing, review and editing. **Wanjun Liu:** conceptualization, formal analysis, methodology, supervision, funding acquisition, review and editing. All authors approved the final version of the manuscript and agreed their accountability in ensuring that any questions related to the accuracy or integrity of any part of the work are appropriately investigated and resolved.

## Ethics Statement

Institutional Ethics Committee of Shanghai Sixth People's Hospital Affiliated to Shanghai Jiao Tong University School of Medicine 2023‐KY‐071(K)‐(1).

## Conflicts of Interest

The authors declare no conflicts of interest.
